# An Account of the Effects of the Astragalus Exscapus Linn. In the Cure of the Venereal Disease

**Published:** 1788

**Authors:** A. Crichton


VII. *An account of the effects of the Astragalus exscapus* Linn. *in the cure of the Venereal Disease; in a letter* from* A. Crichton, *M. D. to* Chr. Girtanner, *M. D. corresponding member of the Royal Society at Gottingen. Translated from the German.*

AS you seem, my dear friend, very anxious to get information concerning the use and effects of the astragalus exscapus, and have requested me to send you my notes of the cases in which I saw it employed, during my stay at Vienna, I communicate them to you with great pleasure. I am fearful, however, that the cases will not appear to you sufficiently accurate, as I had not time to write down the daily changes produced by the remedy, and have omitted to mention in my journal the temperament and habit of body of the several patients. You will find nothing more than their ages, their symptoms at the time of their admission into the hospital, the length of time the medicine was employed, and the effects which might fairly be attributed to its use.

* This letter is extracted from the first volume of a treatise on the venereal disease, by Dr. Girtanner, lately published at Gottingen. See the catalogue of books.

Before I give you these brief notes of the cases in question, permit me first to premise a few observations on the history of the remedy, on the circumstances that led to a more accurate trial of its effects, and the manner in which it was employed in all the instances here related.

This plant, till of late, was equally unknown to physicians and botanists. Linnæus, it is true, mentions it, but without describing it, having never seen the plant. Dr. Jacquin, professor of botany at Vienna, means, however, as he informed me in a conversation I had with him lately (in May 1787) on this subject, soon to publish a figure and description of this plant*.

So far as I can learn it was professor Winterl, at Pest, who first made known the antivenereal powers of this remedy. Some years ago he wrote word to his friends in Vienna, that on the borders of Hungary a domestic

* In a fasciculus of Professor Jacquins's *Icones Plantarum rariorum*, published since the date mentioned by Dr. Crichton, we find a figure of the *astragalus exscapus*, (which is copied by Dr. Girtanner in the work from which the present article is extracted) with a reference to the second volume of his collectanea, in which we may expect a botanical description of the plant. EDITOR.

remedy remedy, which conſiſted of a decoction of the aſtragalus exſcapus, was employed with a very good effect, in the cure of the venereal diſeaſe. Baron Stoerck, on hearing this, was deſirous that Dr. Quarin, as director of the general hoſpital at Vienna, ſhould make ſome experiments with this plant, in order to aſcertain its effects with greater accuracy. A ſhort account of the firſt three caſes in which it was tried, is to be found in the book lately publiſhed by Dr. Quarin, under the title of *Animadverſiones practicæ in diverſos morbos.*

In the ſelection of patients, for theſe experiments, ſuch only were admitted as declared, that before their coming to the hoſpital they had made uſe of no remedy for the venereal diſeaſe; but in caſes of this ſort, in hoſpitals, as you well know, we cannot truſt implicitly to the aſſertions of the patients.

They were all of them under the care of Drs. Quarin and Zeller; and where no contra-indications were preſent, were allowed a full and nouriſhing diet. The apartment in which they were placed was large, and lofty, and the air was frequently renewed. In the winter it was kept warm by means of a ſtove; and during the treatment the patients were not permitted to go out of the room.

In

In all theſe caſes the aſtragalus was preſcribed in the manner following:

℞. Radicis Aſtragali exſcapi unc. ſemiſſ.

Coque in aquæ puræ unc. quindecim, donec libra ſuperſit. Sumat aeger tepide mane, ac veſperi.

Of the following hiſtories of caſes the firſt four are copied from the regiſter belonging to the hoſpital; of the other ſix I was myſelf an eye-witneſs.

### CASE I.

A woman, forty years old, was admitted into the hoſpital, July 26, 1785. She had two venereal nodes on the left parietal bone, and both were in a ſtate of ulceration. She had alſo another conſiderable node on the left tibia, but which was not yet ulcerated. She took a doſe of purgative ſalt, and afterwards began with the aſtragalus exſcapus, prepared in the manner above deſcribed. She continued the uſe of this medicine morning and evening, till the firſt of September, when ſhe was diſcharged from the hoſpital, cured. The two ulcerated nodes, during the treatment, were dreſſed ſimply with digeſtive ointment.——

The

The node on the tibia gradually diminiſhed, and had entirely diſappeared before ſhe quitted the hoſpital. The patient ſweated profuſely during the whole of the time ſhe took the medicine.

### CASE II.

A woman, twenty years old, was admitted into the hoſpital September the 20th, 1785. She had two venereal nodes, one on the tibia, the other on the forehead, but neither of them was ulcerated. She left the hoſpital on the 5th of December following, thoroughly cured; and during this time had taken nothing but the decoction of aſtragalus. At firſt it purged her violently, but this effect ſoon went off; and afterwards, till the end of the treatment, ſhe voided a greater quantity of urine than uſual.

### CASE III.

A woman, twenty years old, was admitted into the hoſpital, October the 6th, 1785. She had a venereal tophus on one tibia, and venereal blotches on the face. On the 19th of November, of the ſame year, ſhe was diſcharged, cured. This patient likewiſe took nothing thing but the aftragalus; and it feemed to increafe the fecretion of urine confiderably.

### CASE IV.

A girl, fifteen years old, was admitted into the hofpital, May 14, 1786. She had a gonorrhœa, and two tophi on the tibia of one foot. She took a faline purge, and afterwards the decoction of aftragalus, without any other remedy. She perfevered in its ufe till the 18th of July, on which day fhe was difcharged, thoroughly cured.

### CASE V.

A woman, twenty-nine years old, came into the hofpital, November 29, 1786, with a gonorrhœa, two large condylomata on the labia pudendi, two buboes, and a tophus on the os frontis. She immediately began taking the decoction of aftragalus, and during the firft three weeks it fweated her profufely. After this fhe got the itch; but this did not prevent her continuing the ufe of the aftragalus till January 29, 1787, on which day fhe was difcharged, cured.

### CASE VI.

Magdalena Jaeger, eighteen years old, was admitted into the hoſpital, on the 25th of January, 1787. She had a gonorrhœa, condylomata on the labia pudendi, ſwelling of the inguinal glands, and the itch. She began the uſe of the decoction of aſtragalus the evening ſhe was admitted, and continued it till the firſt of March following, when ſhe left the hoſpital perfectly cured. She ſweated profuſely during the cure. She took no other medicine than the aſtragalus.

### CASE VII.

Suſanna Caton, thirty-ſeven years old, came into the hoſpital, February 8, 1787. She had a gonorrhœa, and a ſmall node on the os frontis. After eight days uſe of the aſtragalus, the node was almoſt entirely gone, but the gonorrhœa remained as before. She ſweated profuſely, and the ſweat had a ſtrong and diſagreeable ſmell. Some days after this ſhe contracted the itch; but ſhe ſtill continued the uſe of the decoction of aſtragalus, and both the gonorrhœa and the itch ſeemed to be better, as ſhe was diſcharged May 2, at her own requeſt.

### CASE VIII.

Joſeph Koening, aged twenty-ſeven years, came into the hoſpital, April 26, 1787. He had a large, painful tophus on the radius of the left arm, which was already in part ulcerated; another ulcerated tophus on the middle of the eighth rib of the left ſide, and a third on the left clavicle. He immediately began with the decoction of aſtragalus, and ſome of it was applied alſo to the ulcers. Before the 21ſt of June the tophi were thoroughly gone, and the ulcers healed, ſo that he was diſmiſſed cured. In this caſe, during the treatment, the urinary ſecretion was greatly increaſed.

### CASE IX.

Anna Straffer, forty-four years old, came into the hoſpital, April 18, 1787. Her face and head were completely covered with a dry, brown, ſcaly eruption, which, from her own account, and from her mode of life, were ſuppoſed to be venereal. She had likewiſe a gonorrhœa. She continued the ufe of the aftragalus for feveral weeks, without the leaft diminution of the eruption, altho' the gonorrhœa gradually went off. Her hair was now cut off, and her head covered with cloths dipped in a decoction of aftragalus. A flight fuppuration took place under the fcales, which were foft, and gradually fell off; and fhe left the hofpital, cured, June the 4th, 1787.

When one reads thefe cafes, and obferves the excellent effects the aftragalus produced in them, it feems to be a very valuable remedy. In not one of them did it prove inefficacious. There are, however, at Vienna, fome phyficians who have not been equally fuccefsful with it in their experiments; and Profeffor Hunczowfky has affured me, that in his own private practice he has ufed a confiderable quantity of this root, but has feen no good effects from it, and therefore has never perfevered in its ufe longer than a fortnight.

In one cafe I myfelf have feen it adminiftered without any effect. Still this proves, in my opinion, nothing againft the antivenereal power of this remedy; for I am convinced, that in this cafe the principal fymptoms, at the time the aftragalus was had recourse to, were not so much owing to the venereal virus, as to an improper use of mercury.

### CASE X.

A woman, forty years old, was admitted into the hospital, in the beginning of December, 1786. The tonsils and uvula were entirely destroyed; the bones of the nose were carious, and the sores discharged a thin, ill-conditioned, bloody matter. She had been twice salivated, and during the salivation the extent of the ulcers had diminished. By the use of the decoction of astragalus she seemed at first to mend, as the ulcers looked better and redder, and began to heal. But these good effects were not long apparent, and the disease after this seemed to be at a stand, or at least mended extremely slowly, till the end of August, 1787, at which time I quitted Vienna.

The astragalus, besides its anti-venereal properties, is said to possess others which render it very important to physicians, but which I have had no opportunity of ascertaining by my own experience, namely, its good effects in rheumatic, and (sometimes very improperly so called) gouty pains. Professor Hunczowsky, who, as

I have already obferved, will allow no antivenereal property to the aftragalus, affures me, however, that in complaints of the kind juft now mentioned, he has often experienced the moft ftriking good effects from the ufe of this remedy.

This is all I have feen or heard of, for or againft the effects of the aftragalus exfcapus; and I have no doubt but you will agree with me in opinion, that this new remedy, altho' it may not be an actual fpecific for the lues venerea, deferves, however, to be confidered as a valuable addition to the Materia Medica.

---

